# The Familial dementia gene *ITM2b/BRI2* facilitates glutamate transmission via both presynaptic and postsynaptic mechanisms

**DOI:** 10.1038/s41598-019-41340-9

**Published:** 2019-03-19

**Authors:** Wen Yao, Tao Yin, Marc D. Tambini, Luciano D’Adamio

**Affiliations:** Department of Pharmacology, Physiology & Neuroscience New Jersey Medical School, Brain Health Institute, Jacqueline Krieger Klein Center in Alzheimer’s Disease and Neurodegeneration Research, Rutgers, The State University of New Jersey, 185 South Orange Ave, Newark, NJ 07103 USA

## Abstract

Mutations in the *Integral membrane protein 2B* (*ITM2b/BRI2*) gene, which codes for a protein called BRI2, cause familial British and Danish dementia (FBD and FDD). Loss of BRI2 function and/or accumulation of amyloidogenic mutant BRI2-derived peptides have been proposed to mediate FDD and FBD pathogenesis by impairing synaptic Long-term potentiation (LTP). However, the precise site and nature of the synaptic dysfunction remain unknown. Here we use a genetic approach to inactivate *Itm2b* in either presynaptic (CA3), postsynaptic (CA1) or both (CA3 + CA1) neurons of the hippocampal Schaeffer-collateral pathway in both female and male mice. We show that after CA3 + CA1 *Itm2b* inactivation, spontaneous glutamate release and AMPAR-mediated responses are decreased, while short-term synaptic facilitation is increased. Moreover, AMPAR-mediated responses are decreased after postsynaptic but not presynaptic deletion of *Itm2b*. In contrast, the probability of spontaneous glutamate release is decreased, while short-term synaptic facilitation is increased, primarily after presynaptic deletion of *Itm2b*. Collectively, these results indicate a dual physiological role of *Itm2b* in the regulation of excitatory synaptic transmission at both presynaptic termini and postsynaptic termini and suggest that presynaptic and postsynaptic dysfunctions may be a pathogenic event leading to dementia and neurodegeneration in FDD and FBD.

## Introduction

The conditions known as FDD and FBD are due to autosomal dominant mutations in the *ITM2b* gene^1,2^. *ITM2b* codes for the type II membrane protein BRI2. BRI2 is synthesized as a precursor (immature, imBRI2) that is cleaved at the C-terminus by proprotein convertase to produce the mature BRI2 protein (mBRI2) and a 23 amino acid-long (Bri23) soluble C-terminal fragment^3^. In FBD patients, a point mutation at the stop codon of *BRI2* results in a read-through of the 3′-untranslated region and the synthesis of a BRI2 molecule containing 11 extra amino acids at the COOH-terminus. In the Danish kindred, the presence of a 10 nucleotide duplication one codon before the normal stop codon produces a frameshift in the BRI2 sequence generating a precursor protein 11 amino acids larger-than-normal. Convertase-mediated cleavage of mutant British and Danish BRI2 precursor proteins generates two distinct 34 amino acid long peptides, called ABri and ADan, respectively, which are deposited as amyloid fibrils.

BRI2 is a physiological interactor of Aβ-precursor protein (APP)^4,5^, a gene associated with Alzheimer disease^6^. BRI2 binds APP in a region that contains cleavage sites for β-, α- and γ-secretases thereby hindering access to and cleavage of APP by these secretases^7,8^. Analysis of FDD_KI_ and FBD_KI_ mice, two knock-in mouse models of FDD and FBD that carry one mutant and one wild-type *Itm2b* allele, has shown that the Danish and British mutations cause the loss of Bri2 protein, LTP deficits and memory impairments; interestingly, these alterations are APP-dependent^9–15^. Mice carrying one null *Itm2b/Bri2* have similar deficits^10^.

These studies suggest that FDD and FBD may be caused by loss of BRI2 function and increased APP processing and that LTP deficits caused by the loss of Bri2 may be a cellular precursor of dementia. To determine whether Bri2 has a direct synaptic function, and to determine the precise synaptic site of Bri2 function, we performed a systematic genetic analysis by comparing the synaptic effect of global *Itm2b* inactivation to those caused by restricting *Itm2b* inactivation to hippocampal CA1 or CA3 neurons. This strategy allows examination of the effects of simultaneous *Itm2b* inactivation in both presynaptic and postsynaptic neurons as well as selective *Itm2b* inactivation in either presynaptic or postsynaptic neurons of the Schaeffer-collateral pathway.

## Results

### Loss of Bri2 alters excitatory synaptic transmission at hippocampal SC–CA3> CA1 synapses

**Figure 1:**
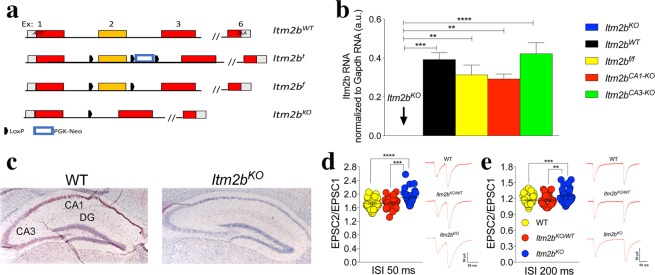
Loss of Bri2 increases synaptic facilitation at hippocampal SC–CA3 > CA1 synapses. (**a**) Schematic representation of the strategy used to generate mice carrying the *Itm2b*^*KO*^ and *Itm2b*^*f*^ alleles. Boxes represent exons (exons 4 and 5 are omitted to save space): coding regions are in red, 5′ and 3′ untranslated regions are in grey. LoxP and PGK-Neo are also depicted. (**b**) RT-PCR shows that *Itm2b* messenger RNA is undetectable in *Itm2b*^*KO*^ mice (n = 3, 2 females and one male) and that *Itm2b*^*f/f*^ (n = 5, 3 males and 2 females), *Itm2b*^*CA1-KO*^ (n = 5, 3 males and 2 females) and *Itm2b*^*CA3-KO*^ (n = 5, 3 males and 2 females) (see experiments shown in Fig. 4 for a description of these mouse lines) mice express *Itm2b* mRNA levels similar to WT animals (n = 5, 3 males and 2 females). Data represent means ± SEM. Data were analyzed by Ordinary one-way ANOVA. ANOVA summary of RT-PCR: F = 11.65, adjusted P value < 0.0001 (significant = ****). Post-hoc Tukey’s multiple comparisons test: WT vs. *Itm2b*^*KO*^ adjusted P value = 0.0001 (significant = ***); *Itm2b*^*f/f*^ vs. *Itm2b*^*KO*^ adjusted P value = 0.0014 (significant = **); *Itm2b*^*CA1-KO*^ vs. *Itm2b*^*KO*^ adjusted P value = 0.0028 (significant = **); *Itm2b*^*CA3-KO*^ vs. *Itm2b*^*KO*^ adjusted P value < 0.0001 (significant = ****); WT vs. *Itm2b*^*f/f*^ adjusted P value = 0.6574 (not significant); WT vs. *Itm2b*^*CA1-KO*^ adjusted P value = 0.443 (not significant); WT vs. *Itm2b*^*CA3-KO*^ adjusted P value = 0.9841 (not significant); *Itm2b*^*f/f*^ vs. *Itm2b*^*CA1-KO*^ adjusted P value = 0.9959 (not significant); *Itm2b*^*f/f*^ vs. *Itm2b*^*CA3-KO*^ adjusted P value = 0.3605 (not significant); *Itm2b*^*CA1-KO*^ vs. *Itm2b*^*CA3-KO*^ adjusted P value = 0.2074 (not significant). (**c**) *In situ* hybridization shows loss of *Itm2b* messenger RNA in all hippocampal cells in *Itm2b*^KO^ mice. Neurons are stained in blue with Haemotoxylin and Eosin, *Itm2b* messenger RNA is stained in red. (**c**) Average PPF (2nd EPSP/1st EPSP) at 50 milliseconds (ms) (number of recordings were: 40, 30 and 36 for *Itm2b*^*WT/WT*^, *Itm2b*^*KO/WT*^ and *Itm2b*^*KO*^ mice, respectively) and 200 ms (number of recordings were: 32, 21 and 30 for *Itm2b*^*WT/WT*^, *Itm2b*^*KO/WT*^ and *Itm2b*^*KO*^ mice, respectively) of the inter-stimulus intervals (ISI). Representative traces of evoked EPSCs are shown (traces are averaged from 20 sweeps). All data represent means ± SEM. Data were analyzed by Ordinary one-way ANOVA. ANOVA summary of 50 ms ISI: F = 11.68, adjusted P value < 0.0001 (significant = ****). Post-hoc Tukey’s multiple comparisons test: WT vs. *Itm2b*^*KO/WT*^ adjusted P value = 0.9562 (not significant); WT vs. *Itm2b*^*KO*^ adjusted P value < 0.0001 (significant = ****); *Itm2b*^*KO/WT*^ vs. *Itm2b*^*KO*^ adjusted P value = 0.0006 (significant = ***). ANOVA summary of 200 ms ISI: F = 9.611, adjusted P value < 0.001 (significant = ***). Post-hoc Tukey’s multiple comparisons test: WT vs. *Itm2b*^*KO/WT*^ adjusted P value = 0.9976 (not significant); WT vs. *Itm2b*^*KO*^ adjusted P value = 0.0004 (significant = ***); *Itm2b*^*KO/WT*^ vs. *Itm2b*^*KO*^ adjusted P value = 0.0014 (significant = **).

*Itm2b* knock-out (*Itm2b*^*KO*^) and floxed *Itm2b* (*Itm2b*^*f/f*^) mice were derived as described previously^7^. Briefly, to generate these animals we targeted *Itm2b* exon 2 because it encodes for the transmembrane region and the proximal part of the extra cellular region of Bri2, which is involved in APP interaction^4,8^. Using a homologous recombination approach, we placed a loxP site ~200 bp 5′ of *Itm2b* exon 2. A *PGK-neo* selection cassette, which contains a neomycin-resistance gene under the control of the *PGK* promoter, surrounded by a 5′ and a 3′ loxP site, has been inserted ~200 bp 3′ of exon 2. This allele is called targeted (*t*) *Itm2b* allele (*Itm2b*^*t*^, Fig. 1A). The rationale for the use of the floxed *PGK-neo* positive selection cassette is the ability to remove the selection cassette by Cre-mediated recombination, eliminating the possibility that presence of the cassette might affect expression of the targeted locus or neighboring genes. Mice expressing a wild-type (WT) and a targeted *Itm2b* allele (*Itm2b*^*t/WT*^) have been crossed to *Meu40*-Cre mice to obtain *Meu40-Cre*/*Itm2b*^*t/WT*^ mice. The Meu40-Cre mouse expresses a mosaic cre-recombinase upon doxaycycline administration, which mediates loxP recombination with low efficiency in gametes. Therefore, in some gametes the recombination of the loxP site flanking the *PGK-neo* cassette will eliminate only the drug resistance gene. These cells will have a floxed *Itm2b* allele in which two loxP sites flank exon 2 (*Itm2b*^*f*^ in Fig. 1a). In other cells the exon 2 region and the *PGK-neo* cassette will be eliminated because of a recombination between the loxP 5′ of exon 2 and 3′ of *PGK-neo*. This total recombination will produce the *Itm2b*^*KO*^ allele (Fig. 1a). Some cells in which the loxP 5′ of exon 2 and 5′ of *PGK-neo* have recombined or in which no recombination occurs will also be generated. Thus, crossing *Meu40-Cre*/*Itm2b*^*t/WT*^ mice to C57BL/6 J yielded *Itm2b*^*f/WT*^ and *Itm2b*^*KO/WT*^ mice.

Female and male *Itm2b*^*KO/WT*^ mice were crossed to generate WT (*Itm2b*^*WT*^), heterozygous (*Itm2b*^*KO/WT*^) and homozygous (*Itm2b*^*KO*^) littermates. *Itm2b*^*KO*^ mice are a whole body *Itm2b* knock out model. Loss of Bri2 protein expression in the brains of these animals was previously tested by Western blot analyses of total brain protein lysates^7^. To test whether *Itm2b*^*KO*^ mice lacked *Itm2b* mRNA expression in total brain and hippocampal neurons, we performed quantitative RT-PCR analysis and *in situ* hybridization experiments. For quantitative RT-PCR analysis we used a probe that encompassed exon 2, the exon deleted by Cre-recombination of the floxed *Itm2b* allele. For the same reason, we used an antisense RNA probe encompassing exon 2 for the *in-situ* hybridization. RT-PCR showed that *Itm2b*^*KO*^ mice had undetectable levels of *Itm2b* mRNA (Fig. 1b). The *in-situ* hybridization experiment confirmed the loss of *Itm2b* mRNA expression in all hippocampal neurons in *Itm2b*^*KO*^ mice (Fig. 1c): thus, *Itm2b*^*KO*^ mice can be used to examine the role of Bri2 at hippocampal SC–CA 3 > CA1 synapses.

Synaptic transmission was studied in 4 to 6-month-old *Itm2b*^*WT*^ (9 males and 9 females), *Itm2b*^*KO/WT*^ (8 males and 8 females) and *Itm2b*^*KO*^ (8 males and 8 females) littermates. First, we examined the effect of *Itm2b* inactivation on paired-pulse facilitation (PPF), a form of short-term synaptic plasticity. PPF is determined, at least in part, by changes in release Probability (P*r*) of glutamatergic synaptic vesicles (SV), such that a decrease in P*r* leads to an increase in facilitation and vice versa^16^. PPF was significantly increased in *Itm2b* deficient mice (Fig. 1d,e), suggesting that Bri2 can tune up glutamate release. To test whether these changes could be caused by differences in time course of recovery (Tau) of the evoked paired-pulse responses EPSCs we quantified the Tau of the evoked PPR EPSCs in Fig. 1d and e there is no significant difference between these groups.(Fig. 1d: *Itm2b*^*WT*^: 53.92 ± 3.85 ms, p = 0.97 compared with *Itm2b*^*KO/WT*^: 51.89 ± 4.03 ms; p = 0.86 compared with *Itm2b*^*KO*^: 58.26 ± 6.55; p = 0.65, *Itm2b*^*KO/WT*^ compared with *Itm2b*^*KO*^; Fig. 1e: *Itm2b*^*WT*^: 52.53 ± 5.18 ms, p = 0.52 compared with *Itm2b*^*KO/WT*^: 45.81 ± 1.96 ms; p = 0.44 compared with *Itm2b*^*KO*^: 45.17 ± 4.24; p = 0.99, *Itm2b*^*KO/WT*^ compared with *Itm2b*^*KO*^; one-way ANOVA).

**Figure 2:**
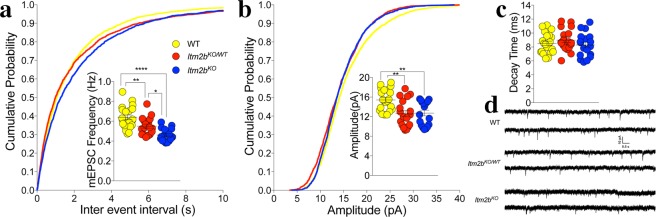
Loss of Bri2 decreases mEPSC frequency and amplitude at hippocampal SC–CA3 > CA1 synapses. (**a**) Cumulative probability of a-amino-3-hydroxy-5-methyl-4- isoxazolepropionic acid receptor (AMPAR) mediated mEPSC inter-event intervals. Inset in cumulative probability graphs represents average mEPSC frequency. (**b**) Cumulative probability of AMPAR-mediated mEPSC amplitudes. Inset in cumulative probability graphs represents average amplitudes. (**c**) Decay time of mEPSC. (**d**) Representative traces of mEPSCs. Number of mEPSCs recordings were: 21, 18 and 18 for *Itm2b*^*WT/WT*^, *Itm2b*^*KO/WT*^ and *Itm2b*^*KO*^ mice, respectively All data represent means ± SEM. Data were analyzed by Ordinary one-way ANOVA. ANOVA summary of mEPSC frequency: F = 19.11, adjusted P value < 0.0001 (significant = ****). Post-hoc Tukey’s multiple comparisons test: WT vs. *Itm2b*^*KO/WT*^ adjusted P value = 0.0096 (significant = **); WT vs. *Itm2b*^*KO*^ adjusted P value < 0.0001 (significant = ****); *Itm2b*^*KO/WT*^ vs. *Itm2b*^*KO*^ adjusted P value = 0.9935 (not significant). ANOVA summary of mEPSC amplitude: F = 8.1, adjusted P value = 0.0008 (significant = ***). Post-hoc Tukey’s multiple comparisons test: WT vs. *Itm2b*^*KO/WT*^ adjusted P value = 0.0027 (significant = **); WT vs. *Itm2b*^*KO*^ adjusted P value = 0.0038 (significant = **); *Itm2b*^*KO/WT*^ vs. *Itm2b*^*KO*^ adjusted P value = 0.8245 (not significant). ANOVA summary of mEPSCs decay time: F = 1.011, adjusted P value = 0.3772 (not significant).

To further test the role of Bri2 in glutamate release, we analyzed miniature excitatory postsynaptic currents (mEPSC). The frequency of mEPSC is also in part determined by changes in P*r*, such that a decrease in P*r* leads to a decrease in frequency and vice versa. The frequency of mEPSC was reduced in *Itm2b* deficient mice in a gene-dosage-dependent manner (Fig. 2a), in accord with the hypothesis that endogenous Bri2 facilitates glutamate release.

**Figure 3:**
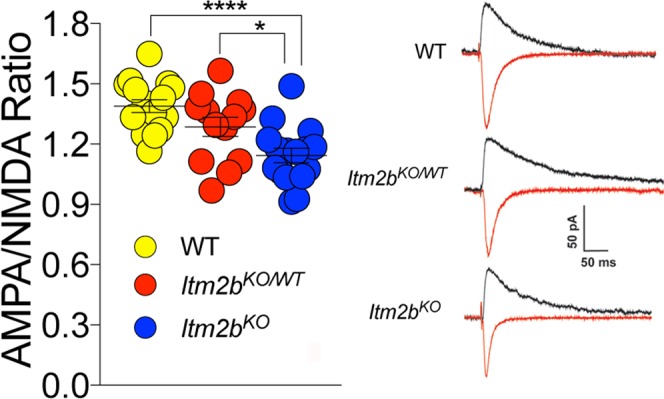
Loss of Bri2 decreases the AMPA/NMDA ratio at hippocampal SC–CA3 > CA1 synapses. AMPA/NMDA ratio is significantly reduced in *Itm2b*^*KO*^ mice. Number of recordings were: 16, 13 and 16 for *Itm2b*^*WT/WT*^, *Itm2b*^*KO/WT*^ and *Itm2b*^*KO*^ mice, respectively. Representative traces of AMPA and NMDA responses are shown on the right of the graph. All data represent means ± SEM. Data were analyzed by Ordinary one-way ANOVA. ANOVA summary, F = 11.13, adjusted P value = 0.0001 (significant = ***). Post-hoc Tukey’s multiple comparisons test: WT vs. *Itm2b*^*KO/WT*^ adjusted P Value = 0.1582 (not significant); WT vs. *Itm2b*^*KO*^ adjusted P Value < 0.0001 (significant = ****); *Itm2b*^*KO/WT*^ vs. *Itm2b*^*KO*^ adjusted P Value = 0.0356 (significant = *).

The amplitude of mEPSCs, which is dependent on postsynaptic AMPA (α-amino-3-hydroxy-5-methyl-4-isoxazole propionic acid) receptor (AMPAR-mediated responses), was significantly decreased in both *Itm2b*^*KO*^ and *Itm2b*^*KO/WT*^ mice (Fig. 2b) while the decay time was unchanged (Fig. 2c). These data suggest that Bri2 boosts the amplitude of AMPAR-mediated responses. To further test whether postsynaptic AMPAR-mediated responses are reduced in *Itm2b* mutant mice, we measured AMPAR and NMDAR-dependent synaptic responses. Consistent with the hypothesis that Bri2 boosts AMPAR-mediated responses, the AMPA/NMDA ratio was reduced in *Itm2b*^*KO*^ (Fig. 3).

### Bri2 plays both a pre and postsynaptic role in excitatory synaptic transmission at hippocampal SC–CA3 > CA1 synapses

**Figure 4:**
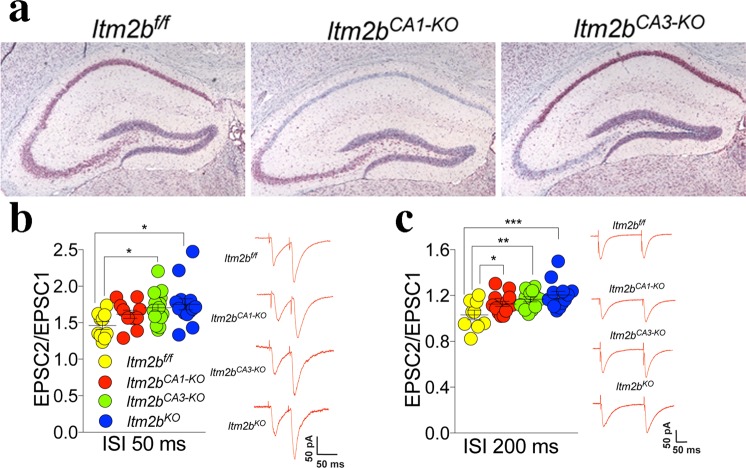
Synaptic facilitation is increased by both pre- and post-synaptic deletion of Bri2. (**a**) *In situ* hybridization shows complete loss of *Itm2b* messenger RNA in CA1 neurons in *Itm2b*^*CA1-KO*^ mice and in CA3 neurons in *Itm2b*^*CA3-KO*^ mice, respectively. *Itm2b*^*f/f*^ mice express *Itm2b* messenger RNA in all hippocampal neurons, just like WT mice, underlying that the LoxP sequences did not alter *Itm2b* expression. Scale bars, 200μm. (**b**) Average PPF at 50 ms ISI. Number of recordings were: 10, 10, 17 and 14 for *Itm2b*^*f/f*^, *Itm2b*^*CA1-KO*^, *Itm2b*^*CA3-KO*^ and *Itm2b*^*KO*^ mice, respectively. Representative traces of evoked EPSCs are shown (traces are averaged from 20 sweeps). (**c**) Average PPF at 200 ms ISI. Number of recordings were: 9, 14, 13 and 13 for *Itm2b*^*f/f*^, *Itm2b*^*CA1-KO*^, *Itm2b*^*CA3-KO*^ and *Itm2b*^*KO*^ mice, respectively. Representative traces of evoked EPSCs are shown (traces are averaged from 20 sweeps). Data were analyzed by Ordinary one-way ANOVA. ANOVA summary of PPF 50 ms ISI: F = 3.902, adjusted P Value = 0.0143 (significant). Post-hoc Tukey’s multiple comparisons test: *Itm2b*^*f/f*^ vs. *Itm2b*^*CA1-KO*^ adjusted P Value = 0.4079 (not significant); *Itm2b*^*f/f*^ vs. *Itm2b*^*CA3-KO*^ adjusted P Value = 0.0377 (significant = *); *Itm2b*^*f/f*^ vs. *Itm2b*^*KO*^ adjusted P Value = 0.0128 (significant = *). *Itm2b*^*CA1-KO*^ vs. *Itm2b*^*CA3-KO*^ adjusted P Value = 0.7363 (not significant); *Itm2b*^*CA1-KO*^ vs. *Itm2b*^*KO*^ adjusted P Value = 0.4416 (not significant); *Itm2b*^*CA3-KO*^ vs. *Itm2b*^*KO*^ adjusted P Value = 0.9322 (not significant). ANOVA summary of PPF 200 ms ISI: F = 5.712, adjusted P value = 0.0021 (significant). Post-hoc Tukey’s multiple comparisons test: *Itm2b*^*f/f*^ vs. *Itm2b*^*CA1-KO*^ adjusted P Value = 0.0306 (significant = *); *Itm2b*^*f/f*^ vs. *Itm2b*^*CA3-KO*^ adjusted P Value = 0.003 (significant = **); *Itm2b*^*f/f*^ vs. *Itm2b*^*KO*^ adjusted P Value = 0.0003 (significant = ***). *Itm2b*^*CA1-KO*^ vs. *Itm2b*^*CA3-KO*^ adjusted P Value = 0.2977 (not significant); *Itm2b*^*CA1-KO*^ vs. *Itm2b*^*KO*^ adjusted P Value = 0.0513 (not significant); *Itm2b*^*CA3-KO*^ vs. *Itm2b*^*KO*^ adjusted P Value = 0.3566 (not significant).

The data above suggest that Bri2 may have both pre- and postsynaptic functions. To directly test this hypothesis, we examined the effects of selective *Itm2b* inactivation in either presynaptic or postsynaptic neurons of the Schaeffer-collateral pathway. To produce CA1- and CA3-restricted *Itm2b* knockout mice (*Itm2b*^*CA1-KO*^ and *Itm2b*^*CA3-KO*^, respectively) we crossed *Itm2b*^*f/f*^ mice (obtained by crossing male and females *Itm2b*^*f/WT*^ mice) to either B6.Cg-Tg(α*CaMKII-cre*) or G32-4 transgenic mice. In the B6.Cg-Tg(α*CaMKII-cre*) line^17^, the mouse α*CaMKII* promoter drives Cre expression in CA1 pyramidal cells, where it drives recombination of the *Itm2b*^*f/f*^ alleles to generate *Itm2b*^*CA1-KO*^ mice. In the G32-4 line^18^, Cre recombinase is under the control of the *Grik4* promoter and is expressed in all CA3 pyramidal cells by 8 weeks of age but is absent in most other brain regions including the CA1. Crossing G32-4 to *Itm2b*^*f/f*^ mice will generate *Itm2b*^*CA3-KO*^ mice. Total brain *Itm2b* mRNA expression is not altered in one-year old *Itm2b*^*CA1-KO*^ and *Itm2b*^*CA3-KO*^ mice (Fig. 1b). In contrast, *in situ* hybridization showed the loss of *Itm2b* mRNA expression in CA1 and CA3 neurons of *Itm2b*^*CA1-KO*^ and *Itm2b*^*CA3-KO*^ mice, respectively, already at 3 months of age (Fig. 4a). Together, these data confirm that *Grik4-Cre* and α*CaMKII-Cre* drive selective loss of *Itm2b* mRNA expression and that this selectivity is stable and is not lost during aging of mice. As shown in Fig. 1b, *Itm2b*^*f/f*^ mice express levels of *Itm2b* messenger RNA comparable to wild-type mice and express *Itm2b* mRNA in both CA1 and CA3 neurons (Fig. 4a), confirming that LoxP did not alter *Itm2b* expression. Thus, in our experiments we tested the following four genotypes: *Itm2b*^*KO*^, *Itm2b*^*CA1-KO*^, *Itm2b*^*CA3-KO*^ and *Itm2b*^*f/f*^ mice. *Itm2b*^*f/f*^ mice were used as control group since *Itm2b* expression is normal in these mice and the other three groups were derived from *Itm2b*^*f/f*^ animals.

**Figure 5:**
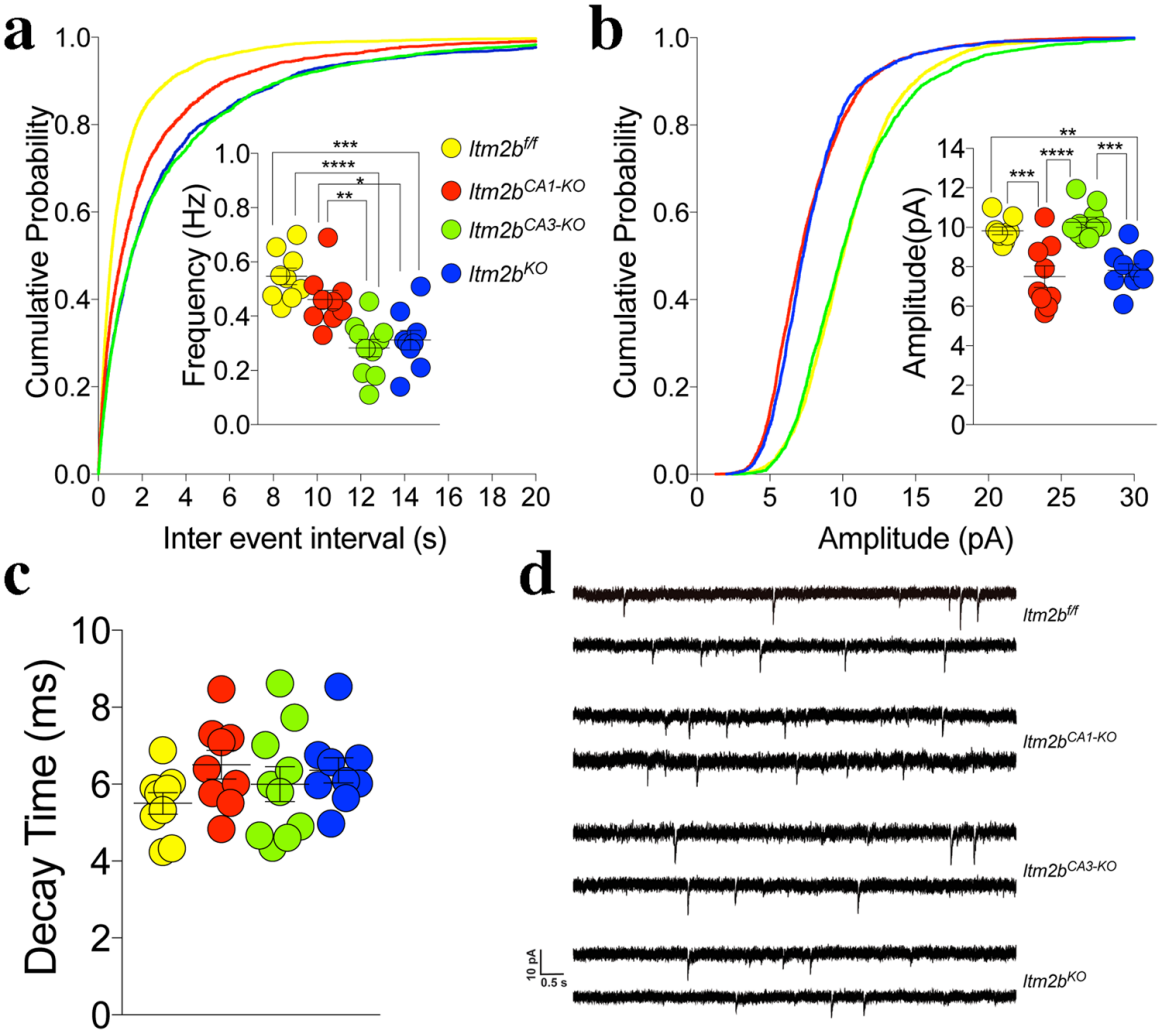
Frequency and amplitude of mEPSCs are reduced by pre- and post-synaptic deletion of Bri2, respectively. (**a**) Cumulative probability of AMPAR mediated mEPSC inter-event intervals. Inset in cumulative probability graphs represents average mEPSC frequency. (**b**) Cumulative probability of AMPAR-mediated mEPSC amplitudes. Inset in cumulative probability graphs represents average amplitudes. (**c**) Decay time of mEPSC. (**d**) Representative traces of mEPSCs. Number of mEPSCs recordings were: 9, 9, 10 and 9 for *Itm2b*^*f/f*^, *Itm2b*^*CA1-KO*^, *Itm2b*^*CA3-KO*^ and *Itm2b*^*KO*^ mice, respectively. Data were analyzed by Ordinary one-way ANOVA. ANOVA summary of mEPSCs frequency: F = 14.46, adjusted P value < 0.0001 (significant = ****). Post-hoc Tukey’s multiple comparisons test: *Itm2b*^*f/f*^ vs. *Itm2b*^*CA1-KO*^ adjusted P Value = 0.2848 (not significant); *Itm2b*^*f/f*^ vs. *Itm2b*^*CA3-KO*^ adjusted P Value < 0.0001 (significant = ****); *Itm2b*^*f/f*^ vs. *Itm2b*^*KO*^ adjusted P Value = 0.001 (significant = ***). *Itm2b*^*CA1-KO*^ vs. *Itm2b*^*CA3-KO*^ adjusted P Value = 0.0025 (significant = **); *Itm2b*^*CA1-KO*^ vs. *Itm2b*^*KO*^ adjusted P Value = 0.0167 (significant = *); *Itm2b*^*CA3-KO*^ vs. *Itm2b*^*KO*^ adjusted P Value = 0.9204 (not significant). ANOVA summary of mEPSCs amplitude: F = 15.43, adjusted P value < 0.0001 (significant = ****). Post-hoc Tukey’s multiple comparisons test: *Itm2b*^*f/f*^ vs. *Itm2b*^*CA1-KO*^ adjusted P Value = 0.0004 (significant = ***); *Itm2b*^*f/f*^ vs. *Itm2b*^*CA3-KO*^ adjusted P Value = 0.8203 (not significant); *Itm2b*^*f/f*^ vs. *Itm2b*^*KO*^ adjusted P Value = 0.0022 (significant = **). *Itm2b*^*CA1-KO*^ vs. *Itm2b*^*CA3-KO*^ adjusted P Value < 0.0001 (significant = ****); *Itm2b*^*CA1-KO*^ vs. *Itm2b*^*KO*^ adjusted P Value = 0.9203 (not significant); *Itm2b*^*CA3-KO*^ vs. *Itm2b*^*KO*^ adjusted P Value = 0.0001 (significant = ***). ANOVA summary of mEPSCs decay time: F = 1.419, adjusted P value = 0.255 (not significant).

**Figure 6:**
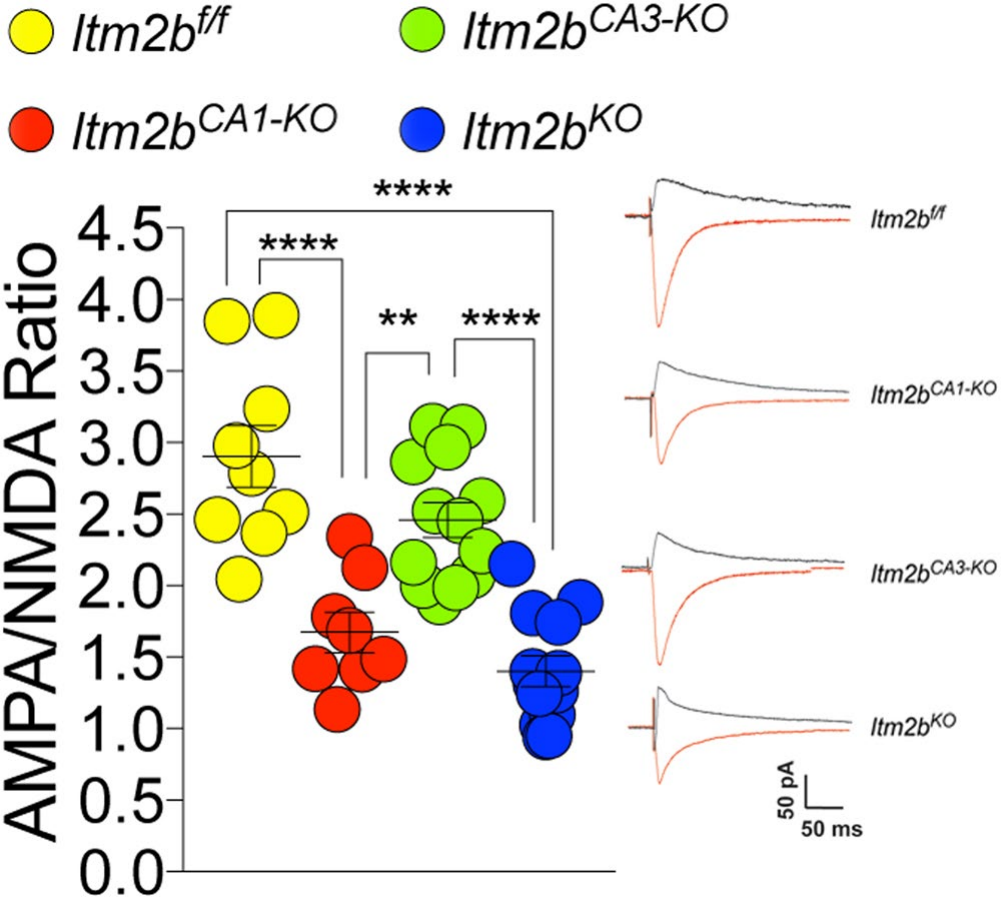
Post-synaptic loss of Bri2 decreases the AMPA/NMDA ratio at hippocampal SC–CA3 > CA1 synapses. AMPA/NMDA ratio is significantly reduced in *Itm2b*^*KO*^ and *Itm2b*^*CA3-KO*^ mice. AMPAR/NMDAR ratio. Number of recordings were: 9, 8, 13 and 13 for *Itm2b*^*f/f*^, *Itm2b*^*CA1-KO*^, *Itm2b*^*CA3-KO*^ and *Itm2b*^*KO*^ mice, respectively. Data were analyzed by Ordinary one-way ANOVA. ANOVA summary of AMPAR/NMDAR ratio: F = 23.08, adjusted P value < 0.0001 (significant = ****). Post-hoc Tukey’s multiple comparisons test: *Itm2b*^*f/f*^ vs. *Itm2b*^*CA1-KO*^ adjusted P Value < 0.0001 (significant = ****); *Itm2b*^*f/f*^ vs. *Itm2b*^*CA3-KO*^ adjusted P Value = 0.1477 (not significant); *Itm2b*^*f/f*^ vs. *Itm2b*^*KO*^ adjusted P Value < 0.0001 (significant = ****); *Itm2b*^*CA1-KO*^ vs. *Itm2b*^*CA3-KO*^ adjusted P Value = 0.0034 (significant = **); *Itm2b*^*CA1-KO*^ vs. *Itm2b*^*KO*^ adjusted P Value = 0.5709 (not significant); *Itm2b*^*CA3-KO*^ vs. *Itm2b*^*KO*^ adjusted P Value < 0.0001 (significant = ****).

We next compared the effect of either global or selective *Itm2b* inactivation on PPF. For these experiments, we used 14–16 months-old *Itm2b*^*KO*^, *Itm2b*^*CA1-KO*^, *Itm2b*^*CA3-KO*^ and *Itm2b*^*f/f*^ mice, 5 females and 5 males for each genotype. PPF was significantly increased in *Itm2b*^*KO*^ and *Itm2b*^*CA3-KO*^ mice at both 50 ms (Fig. 4b) and 200 ms ISI (Fig. 4c). The *Itm2b*^*CA1-KO*^ mice had an intermediate phenotype: the PPF at 50 ms ISI was not significantly different from not only control *Itm2b*^*f/f*^ mice but also *Itm2b*^*KO*^ and *Itm2b*^*CA3-KO*^ mice. At 200 ms ISI, PPF was significantly increased as compared to the control group (Fig. 4c). To further test the role of pre- and postsynaptic Bri2 in glutamate release, we analyzed mEPSC. The frequency of mEPSC was reduced in *Itm2b*^*KO*^ and *Itm2b*^*CA3-KO*^ mice (Fig. 5a). *Itm2b*^*CA1-KO*^ animals showed a frequency that was not significantly different from *Itm2b*^*f/f*^ mice, but it was significantly higher than that of both *Itm2b*^*KO*^ (p = 0.0167) and *Itm2b*^*CA3-KO*^ (p = 0.0025) mice. Altogether these data unambiguously underscore a role for presynaptic Bri2 in tuning up glutamate release, and also suggest that postsynaptic Bri2 may play a part in glutamate release. Analysis of the amplitude of mEPSCs shows a significant decrease in mEPSCs amplitude in both *Itm2b*^*KO*^ and *Itm2b*^*CA1-KO*^ mice. In contrast, mEPSCs amplitude is normal in *Itm2b*^*CA3-KO*^ mice (Fig. 5b). Thus, postsynaptic but not presynaptic Bri2 boosts the amplitude of spontaneous glutamatergic responses. Consistent with this hypothesis, the AMPAR/NMDAR ratio was reduced in *Itm2b*^*KO*^ and *Itm2b*^*CA1-KO*^ mice but was normal in *Itm2b*^*CA3-KO*^ animals (Fig. 6). The AMPAR-mediated responses are considerably smaller in this set of experiments as compared to those with the 4–6 months-old animals (Fig. 2). This is most likely due to the fact that amplitudes of mEPSCs are significantly reduced in aged mice^19^. The decrease in the PPR values seen in Fig. 4 as compared to Fig. 1 is likely to be related to age differences between these two cohorts of animals as well.

## Discussion

Collectively, our studies demonstrate that the loss of Bri2 impairs glutamatergic neurotransmitter release and AMPAR-mediated responses by a presynaptic and postsynaptic mechanism, respectively. Simultaneous *Itm2b* inactivation in both CA3 and CA1 neurons leads to increased PPF and decreased mEPSC frequency, suggesting that loss of Bri2 causes a reduction in the release probability of glutamate. The changes in glutamate release are principally due to a presynaptic loss of Bri2 since mEPSC frequency was reduced and PPF was increased equally in both *Itm2b*^*KO*^ and *Itm2b*^*CA3-KO*^ mice. However, *Itm2b*^*CA1-KO*^ mice showed an intermediate phenotype between control *Itm2b*^*f/f*^ and *Itm2b*^*KO*^/*Itm2b*^*CA3-KO*^ mice. This observation is counterintuitive since the mechanisms underlying mEPSC frequency and neural facilitation are presynaptic, raising the question of how postsynaptic Bri2 may participate in tuning PPF. Three main possibilities may explain these observations. A) In B6.Cg-Tg*/Itm2b*^*f/f*^ mice, the *αCaMKII-cre* may drive a partial recombination of the floxed *Itm2b* allele in CA3 neurons, which may reduce *Itm2b* mRNA expression sufficiently enough to produce a partial loss of Bri2 function in CA3 neurons in *Itm2b*^*CA1-KO*^ mice, albeit not sufficiently enough to be evident in the *in-situ* hybridization experiments. B) Bri2 is processed by several proteases that release Bri2-derived metabolites extracellularly. Besides the aforementioned convertase-mediated release of Bri23 from imBri2, mBri2 undergoes an additional cleavage by ADAM10 in its ectodomain, which releases a soluble variant of Bri2 into the extracellular space and produces a membrane-bound Bri2 N-terminal fragment. This membrane-bound Bri2 metabolite undergoes intramembrane proteolysis by SPPL2a and SPPL2b to produce an intracellular domain as well as a secreted short C-terminal peptide^20^. Ultimately, Bri2 cleavages can produce at least three distinct secreted Bri2-derived metabolites. Thus, it is possible that one or more of these metabolites produced at postsynaptic termini may play a trans-synaptic physiological role in tuning down PPF. C) Postsynaptic Bri2 could modulate endocannabinoids release from the postsynaptic termini, which in turn could modulate presynaptic functions acting on presynaptic cannabinoid type 1 and type 2 receptors^21^. Further experiments are required to distinguish between these three possibilities.

The amplitude of mEPSC and the AMPAR/NMDAR ratio were both reduced in *Itm2b*^*KO*^ animals, suggesting that endogenous Bri2 is necessary for physiological AMPAR-mediated responses. AMPAR responses are undoubtedly regulated by postsynaptic Bri2, since they were reduced in *Itm2b*^*CA1-KO*^ mice but not *Itm2b*^*CA3-KO*^ animals.

Although future studies are needed to assess the molecular and biochemical mechanisms underlying these pre and postsynaptic functions of Bri2, the evidence presented here may be physiologically and pathologically relevant. In fact, several studies have implicated products of genes mutated in Familial forms of dementia in glutamatergic synaptic transmission. Previous studies investigating synaptic dysfunction induced by Aβ, which derives from APP processing and is considered the main mediator of AD pathogenesis, have suggested that early pathogenic changes in AD were driven by postsynaptic impairments^22–29^. More recently, presynaptic functions of APP that modulate glutamate release have been described^30,31^. The other familial Alzheimer’s proteins PS1 and PS2 regulate glutamate release in mature neurons by a presynaptic mechanism^32–34^. Our findings that a fourth familial dementia protein, Bri2, also physiologically fine-tunes glutamate transmission with both pre- and postsynaptic mechanisms, suggest that defects in excitatory neurotransmitter release may represent a general and convergent mechanism leading to neurodegeneration. In addition, these findings further support the hypothesis, put forward several years ago^10^, that familial Danish, British and Alzheimer’s dementias share a pathogenic sameness and that *Itm2b* should be recognized as a fourth Familial Alzheimer disease gene.

## Methods

### Mice and ethics statement

Mice were handled according to the Ethical Guidelines for Treatment of Laboratory Animals of the NIH. The procedures were described and approved by the Institutional Animal Care and Use Committee (IACUC) at Rutgers. The Newark IACUC is part of Rutgers Office of Research Regulatory Affairs (ORRA), which oversees the conduct of research to promote integrity of the scientific record, including training and certification as appropriate. Rutgers recognizes the vital role of animals in biomedical research. Comparative Medicine Resources (CMR) serves scientists and assures animal well-being. Our staff of veterinarians, technicians and support personnel are dedicated to the humane and ethical use of laboratory animals. Rutgers complies with federal mandates, namely the Animal Welfare Act and Public Health Service Policy and works closely with the IACUC that reviews and regulates the use of animals for biomedical research and teaching. The University has had full accreditation from Association of Assessment and Accreditation of Laboratory Animal Care (AAALAC) International since 1981 and Letters of Assurance are on file with the NIH in the Office of Laboratory Animal Welfare (OLAW). *Itm2b*^*f/f*^ and *Itm2b*^*KO*^ mice were described previously^7^. The B6.Cg-Tg(Camk2a-cre)T29-1Stl/J(Stock No:005359 T29-1) and C57BL/6-Tg(Grik4-cre)G32-4Stl/J (Stock No: 006474) mice were originally purchased from The Jackson Laboratory.

### Brain slice preparation

Mice were deeply anesthetized with isoflurane, and intracardially perfused with an ice-cold cutting solution containing (in mM) 120 Choline Chloride, 2.6 KCl, 26 NaH CO3, 1.25 NaH2PO4, 0.5 CaCl2, 7 MgCl2, 1.3 Ascorbic Acid, 15 Glucose, pre-bubbled with 95% O2/5% CO2 for 15 minutes. The brains were rapidly removed from the skull. Coronal brain slices containing the hippocampal formation (350 μm thick) were prepared in the ice-cold cutting solution bubbled with 95% O2/5% CO2 using Vibratome VT1200S (Leica Microsystems, Germany) and then incubated in an interface chamber in ACSF containing (in mM): 126 NaCl, 3 KCl, 1.2 NaH2PO4; 1.3 MgCl2, 2.4 CaCl2, 26 NaHCO3, and 10 glucose (at pH 7.3), bubbled with 95% O2 and 5% CO2 at 30 °C for 1 hour and then kept at room temperature. The hemi-slices were transferred to a recording chamber perfused with ACSF at a flow rate of ~2 ml/min using a peristaltic pump. Experiments were performed at 28.0 ± 0.1 °C.

### Whole-cell electrophysiological recording

Whole-cell recordings in the voltage-clamp mode(-70 mv) were made with patch pipettes containing (in mM): 132.5 Cs-gluconate, 17.5 CsCl, 2 MgCl2, 0.5 EGTA, 10 HEPES, 4 ATP, and 5 QX-314, with pH adjusted to 7.3 by CsOH. Patch pipettes (resistance, 8–10 MΩ) were pulled from 1.5 mm thin-walled borosilicate glass (Sutter Instruments, Novato, CA) on a horizontal puller (model P-97; Sutter Instruments, Novato, CA).

Basal synaptic responses were evoked at 0.05 Hz by electrical stimulation of the Schaffer collateral afferents using concentric bipolar electrodes. CA1 neurons were viewed under upright microscopy (FN-1, Nikon Instruments, Melville, NY) and recorded with Axopatch-700B amplifier (Molecular Devices, San Jose, CA). Data were low-pass filtered at 2 kHz and acquired at 5–10 kHz. The series resistance (Rs) was consistently monitored during recording in case of reseal of ruptured membrane. Cells with Rs >20 MΩ or with Rs deviated by >20% from initial values were excluded from analysis. Excitatory postsynaptic currents (EPSCs) were recorded in ACSF containing 15 μM bicuculline methiodide to block GABA-A receptors. The stimulation intensity was adjusted to evoke EPSCs that were 40% of the maximal evoked amplitudes (“test intensity”). 5–10 min after membrane rupture, EPSCs were recorded for 7 min at a test stimulation intensity that produced currents of ~40% maximum. For recording of paired-pulse ratio (PPR), paired-pulse stimuli with 50 ms or 200 ms inter-pulse interval were given. The PPR was calculated as the ratio of the second EPSC amplitude to the first. For recording of AMPA/NMDA ratio, the membrane potential was firstly held at-70 mV to record only AMPAR current for 20 sweeps with 20 s intervals. Then the membrane potential was turned to +40 mV to record NMDAR current for 20 sweeps with perfusion of 5 μM NBQX to block AMPAR. Mini EPSCs were recorded by maintaining neurons at −70 mV with ACSF containing 1 μM TTX and 15 μM bicuculline methiodide to block action potentials and GABA-A receptors respectively. mEPSCs were recorded for 5–10 mins for analysis. Data were collected and analyzed using the Axopatch 700B amplifiers and pCLAMP10 software (Molecular Devices) and mEPSCs are analyzed using mini Analysis Program.

### mRNA detection

RT-PCR. Total brain RNA was extracted from 1-year old mice with RNeasy RNA Isolation kit (Qiagen 74104) and used to generate cDNA with a High-Capacity cDNA Reverse Transcription Kit (Thermo 4368814). 50 ng cDNA, TaqMan™ Fast Advanced Master Mix (Thermo 4444556), and the appropriate TaqMan (Thermo) probes were used in the real time polymerase chain reaction. Samples were analyzed on an Applied QuantStudio™ 6 Flex Real-Time PCR System, and relative RNA amounts were quantified using LinRegPCR software (hartfaalcentrum.nl). The probe Mm01310552_mH (exon junction 1–2) was used to detect mouse Itm2b and samples were normalized to Gapdh levels, as detected with Mm99999915_g1 (exon junction 2–3).

*In-situ. Itm2b* mRNA was detected in 3-month mice (WT, *Itm2b*^*f/f*^, *Itm2b*^*KO*^, *Itm2b*^*CA1-KO*^ and *Itm2b*^*CA3-KO*^) using BaseScope (Advanced Cell Diagnostics, Hayward, CA). BA-Mn-Itm2b-E2–3ZZ RNA probe was designed to specifically detect Exon2 of *Itm2b* mRNA (bases 263–384 of NM_008410.2). Mice were perfused with PBS and subsequently with 4% paraformaldehyde at room temperature. The whole brains were dissected and immersed in 4% paraformaldehyde for 48 h at 4 °C. Fixed brains were transferred into 20% sucrose solution, followed by 30% sucrose solution and imbibing into O.C.T Compound. Samples were stored at −80 °C. 12 ηm sections were cut with a cryostat, mounted on Superfrost Plus slides at −20 °C and stored at −80 °C. Brain sections were baked at 60 °C for 45 min and subsequently fixed in fresh 4% paraformaldehyde for 90 min at room temperature. Re-fixed brain sections were dehydrated by gradient ethanol, followed by a 10 min pre-treatment of RNAscope Hydrogen peroxide and a 30 min pre-treatment of RNAscope Protease IV at room temperature (ACD, Ref 322381). Pre-treated sections were incubated with the *Itm2b* probe for 2 h in 40 °C and followed signal detection by BaseScope detection reagent kit-RED (ACD, Ref 322910). Images were captured by OCULAR software with automatic exposure using Nikon Eclipse 50i microscope system at 4x magnification.

### Statistics

Recordings were analyzed by one-way ANOVA. Data that showing statistical significance by one-way ANOVA were subsequently analyzed by Tukey’s multiple comparisons test. All statistical analyses were performed using Prism 7 (GraphPad) software.
